# Intrauterine contraceptive device training and outcomes for healthcare providers in developed countries: A systematic review

**DOI:** 10.1371/journal.pone.0219746

**Published:** 2019-07-15

**Authors:** Menglu Ouyang, Ke Peng, Jessica R. Botfield, Kevin McGeechan

**Affiliations:** 1 The George Institute for Global Health, Faculty of Medicine, University of New South Wales, Sydney, Australia; 2 School of Public Health, University of Sydney, Sydney, Australia; 3 Family Planning NSW, Ashfield, Sydney, Australia; Aga Khan University, KENYA

## Abstract

Intrauterine contraceptive devices (IUCD) are a safe and cost-effective contraceptive method for medically eligible women. Despite this, the utilisation rate for IUCDs is relatively low in many high-income countries, including Australia. Provision of education and training regarding IUCDs to healthcare providers, including nurses and midwives, is one approach to overcome some of the barriers that may prevent wider uptake of IUCDs. This study aims to explore the types and impact of IUCD insertion training for healthcare providers. A systematic review was undertaken in January 2017 to determine the effectiveness of IUCD training for healthcare providers in relation to provision of IUCDs to women. The databases MEDLINE, EMBASE, CINAHL, COCHRANE and SCOPUS were searched to identify studies from high-income countries relating to IUCD training for healthcare providers and relevant outcomes. A total of 30 studies were included in the review. IUCD training for healthcare providers contributed to increased knowledge and improved positive attitudes towards IUCDs, high rates of successful insertions, low complication rates, and increased provision of IUCDs. Successful insertions and low complication rates were similar across different healthcare provider types. No notable differences between provider types in terms of knowledge increase or insertion outcomes were observed. Different training programs for healthcare providers were found to be effective in improving knowledge and successful provision of IUCDs. Increasing the number of healthcare providers skilled in IUCD insertions in high-income countries, including nurses and midwives, will enhance access to this method of contraception and allow women greater contraceptive choice.

## Introduction

Long acting reversible contraceptives (LARCs), including intrauterine contraceptive devices (IUCDs), are methods for preventing unintended pregnancies that do not require daily adherence [1]. The effectiveness of the IUCD as a contraceptive method is approximately 99.2% to 99.8% within the first year of use, which is higher than other shorter-term reversible contraceptive methods, such as the oral contraceptive pill, within the same timeframe of use [2]. Advantages of IUCDs include long-term effectiveness, easily reversible, safety for use in post-abortion patients, and use as emergency contraception [3].

In 2015, the proportion of women choosing IUCDs as a contraceptive method was 21% worldwide [4]. However, the rates of women using IUCDs in some higher income countries are relatively low. For example, the prevalence of IUCD use is 5.2% in the United States of America (USA), 10.3% in the United Kingdom (UK), 13.4% in France and 19.0% in Sweden [5, 6]. In Australia, the proportion of women using IUCDs is just 6.1% [7].

Factors related to IUCD uptake include accessibility of insertion services, users’ awareness of and attitudes to this method, and healthcare providers’ attitudes, knowledge and skills in insertion [6, 8]. Despite the long-term cost-effectiveness of IUCDs, their upfront cost can also be an obstacle for many women [9]. Further to this, health care providers, including primary care physicians and nurses as well as specialists, play an important role in women’s access to and decision-making regarding contraceptive choice and can either restrict or enable uptake of IUCDs [6]. A survey undertaken in Australia to investigate perceived barriers to use of IUCDs by healthcare providers reported the following concerns: difficulties inserting IUCDs (63%), risk of pelvic inflammatory disease (53%), insertion pain (45%) and infertility (34%) [1]. Healthcare providers’ attitudes regarding the appropriateness of IUCDs for some women, including adolescent or nulliparous women, may also limit access [6]. Concerns and misperceptions such as these are likely to impact on the provision and uptake of IUCDs.

One approach advocated to increase access to IUCDs is through the provision of education and training for healthcare providers to increase their knowledge, skills and confidence in discussing and providing this method of contraception [6, 10]. IUCD insertions are primarily undertaken by a general practitioner (GP) or gynaecologist, although nurses with appropriate training may also carry out insertions. The training requirements for nurses are similar across countries such as Australia, the USA and UK, although in Australia, relatively few nurses have been trained to carry out IUCD insertions. This may be in part due to the differences in the funding of these services between countries.

This review sought to identify the different types of education and training on IUCD insertion that have been evaluated and in particular their outcomes on provision of IUCDs. We also sought to assess whether there were any notable differences between healthcare provider type.

## Method

This systematic review was conducted following the Preferred Reporting Items for Systematic Reviews and Meta-analysis (PRISMA) guidelines. A search of peer-reviewed literature was originally undertaken during 23 January and 31 March 2017, using the following search criteria for study selection:

1. Language: English only
2. Database: MEDLINE, EMBASE, CINAHL, COCHRANE and SCOPUS
3. Year: No limit on year of publication
4. Publication: Peer-reviewed research article
5. Countries: High-income countries, as defined by the World Bank [11]
6. Search terms in MEDLINE: (‘exp Intrauterine Devices/’ OR ‘Intrauterine Devices, Copper/’ OR ‘IUCD.mp.’ OR ‘Contraceptive Devices/’ OR ‘IUD.mp.’) AND (‘exp Education/’ OR ‘exp Education, Continuing/’ OR ‘Inservice Training/’ OR ‘insertion training.mp.’ ‘training*.mp.’) AND (‘clinician*.mp.’ OR ‘physician*.mp.’ OR ‘Family Nurse Practitioners/’ OR ‘nurse*.mp.’ OR ‘exp General Practitioners/’ OR ‘Gynaecologist.mp.’ OR ‘Obstetrician.mp.’ OR ‘exp Physicians/’ OR ‘exp Medical Staff/’)

**Fig 1:**
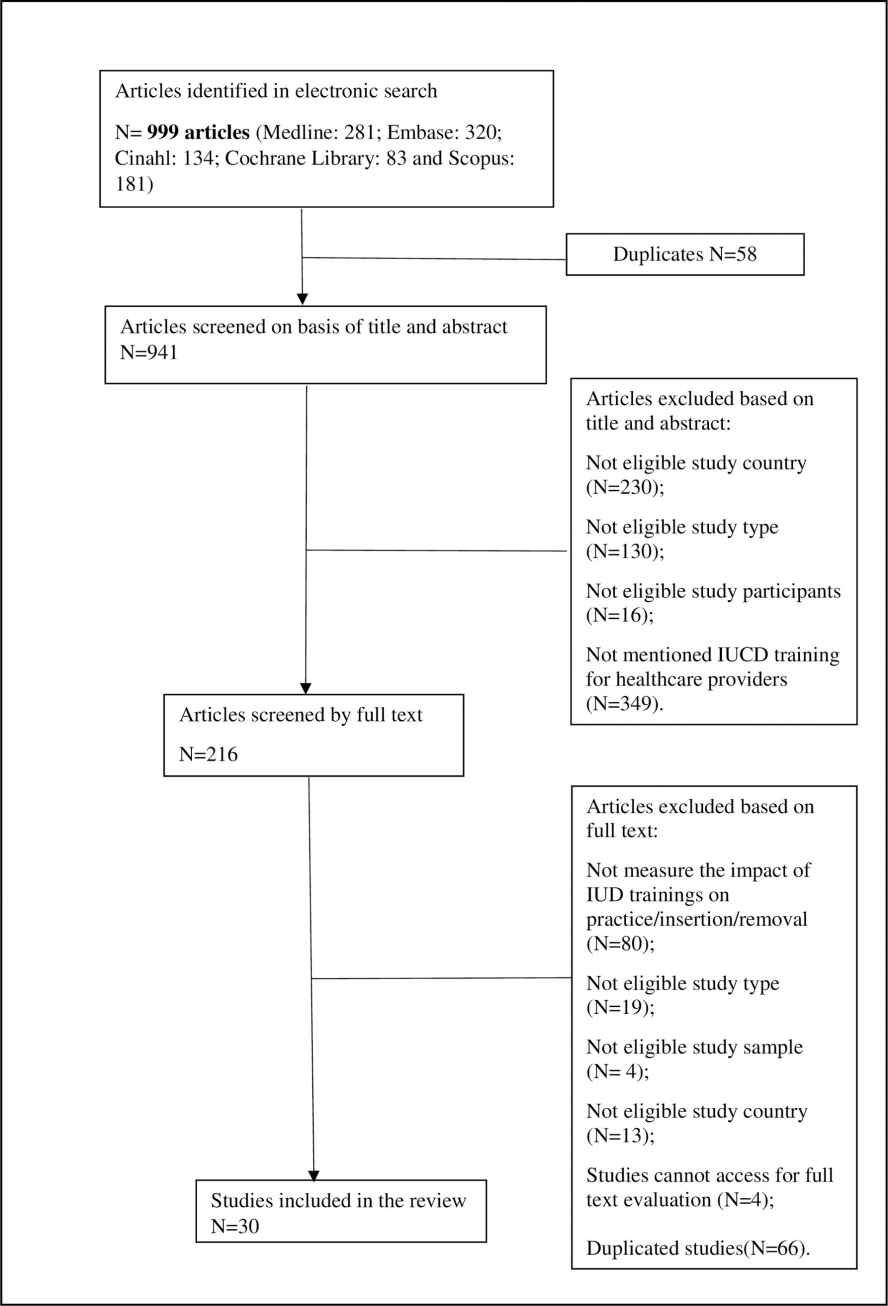
Flow chart of article selection process.

All articles identified in the search were screened by the first author (MO) based on title and abstract for potential inclusion. Authors 1 (MO) and 2 (KP) then independently screened the full-text articles to determine their eligibility for inclusion. Disagreements were resolved by discussion or with a third review (KM). Inclusion criteria were 1) evaluated the IUCD training or clinical education programs for health care providers permitted to insert IUCDs; and 2) study participants included physicians, nurses, midwives, medical officers, general practitioners, gynaecologists and/or obstetricians. There was no limitation on the study designs except general discussions without presentation of data and results. We excluded studies that were conducted in low- or middle- income countries according to the World Bank definition [11] or which did not measure the outcomes of insertion/removal practices of clinicians after IUCD training. A flowchart for the selection of eligible research articles is presented in Fig 1.

After confirming eligibility for inclusion, the following data were extracted—authors, geographical location, study design, study setting, study type and size of participants, year of publication, type/s of IUCD training and outcome details—by the first author and then independently checked by the second author. The GRADE system and risk of bias approach, as recommended by the Cochrane Collaboration, were used to evaluate the quality of evidence of each selected study [12]. As there was heterogeneity among the studies with respect to study design (RCT, cohort or cross-sectional study), study participants and outcomes measured (incidence, frequency or association), summary measures of the effect of IUCD training were not calculated.

## Results

The combined database searches yielded 941 results. Of these, 216 were included to conduct full-text screening, following review of the title and abstract. In reviewing the full text, 30 studies were identified as eligible to include. The quality of evidence of most ranged from very low to moderate, primarily because the majority of studies were observational studies with small sample sizes (Table 1).

**Table 1:** Impact of IUCD training on doctors, nurses and residents on IUCD insertion and adverse effects or other complications.

Affected health care providers	Author (year)	Impact of IUCD training	Level of Evidence
**Doctors**	Stewart M et al. (2016) ^[^^22^^]^	Eighty-nine percent (212/238) insertions were reported to be successful in the follow up, fewer referrals in the 12 months post-training than in the three months pre-training. Two cases of malposition and 5 cases of expelled were reported. No cases of infection or perforation. Participants reported felt more confident in IUD practice after training.	Low
	Thompson KM et al. (2016) ^[^^24^^]^	LARC initiation is higher in intervention group compare to control group (22 vs. 18 per 100 Person Year).	High
	Luchowski AT et al. (2014) ^[^^13^^]^	There is no association between training intervention and number of IUDs inserted in the past year. 70.4% of obstetricians and gynaecologists had inserted at least one copper IUD during residency. Clinicians reported that inserted at least one IUD past year is associated with numbers of IUD inserted during residency.	Low
	Lunde B et al. (2014) ^[^^16^^]^	Family physicians (70.4%) were more likely to receive training in placement of IUDs compared to medicine physicians (9.9%). Most of physicians (86%-90%) had training in IUD placement during residency. Physicians who were trained in IUD placement after residency were more likely to place IUD than those trained during residency (Adjusted OR: 2.1, 95% CI: 1.0–4.5).	Low
	Greenberg KB et al. (2013) ^[^^42^^]^	Women's health training was the strongest predictor of LARC provision. The ORs of obstetricians or family medicine physicians who had residency training are 83.83 times more likely to provision IUDs compare to other clinicians who did not have family planning training.	Very low
	Phillips SP et al. (2010) ^[^^27^^]^	The number of family planning physicians inserted IUDs increased from 11% to 31%. Total number of IUD insertions increased from 250 before intervention to 337 after intervention, compared to 231 and 259 in control group.	Very low
	Madden T et al. (2010) ^[^^39^^]^	Physicians who finished training after year of 1999 or who met a greater number of patients per week were less likely to have low IUD insertion rate compare to those who had training before year of 1998.	Low
	Moss E et al. (2009) ^[^^41^^]^	55% respondents stated they had inserted more than 10 devices while 31% indicated inserted 6 to 10 IUDs per year.	Very low
	Goodman S et al. (2008) ^[^^32^^]^	The mean IUD insertions per month increased from 28 to 71 after trainings. Rate of complication is rare with 5.4% reported in post-abortal insertion and 1.8% in interval insertions.	Low
	Markham WA et al. (2005) ^[^^30^^]^	69% GPs trainers believed that the initial IUD training and re-certification requirements discourage them from IUD insertion training.	Very low
	Richardson A et al. (1993) ^[^^29^^]^	There were 126 insertions (27%) with at least one relative contraindication after training. Gynaecologist performed insertions had fewer relative contraindications (30%) compared to other doctors (48%).	Low
**Nurses**	Kemeny F et al. (2016) ^[^^18^^]^	After training, ninety-one percent (188/207) of insertions by registered nurses were successful. In the 6-month follow up, 2% reported expelled, 1% malposition confirmed and 3% removed but not replaced. No perforations are known to have occurred during insertions.	Low
	Dermish A et al. (2016) ^[^^20^^]^	Fail insertion rate decreased from 12.8% to 4.3% post-intervention. The odds of a successful insertion post-intervention was 4.8 times pre-intervention when adjust confounders. 3 explosions occurred pre-intervention, and 1 uterine perforation post-intervention. 59 of 68(87%) and 42 of 57(74%) were comfortable with their skills immediately after the training at 6 months respectively	Moderate
	Harper CC et al. (2013) ^[^^17^^]^	Practicum training for insertions was significant associated with greater provision later in practice (OR = 2.4, 95%CI: 1.10 to 5.33). Nurse practitioners working in women's health were more likely to receive IUD insertion practicum training compare to those working in primary care (66% vs. 12%). Training increased comfort of inserting IUDs and improved common knowledge of IUDs. Primary care NPs were less likely to routinely counsel on IUDs, and they had lower odds of IU provision	Moderate
	Andrews GD et al. (1999) ^[^^21^^]^	Of 50 IUDs were inserted after training, 22 was inserted by trained nurses. At the 6-weeks follow up remained 29 patients, and only 8 of them reported minor problems (4 inserted by trained nurses and 4 by doctors). 8 IUDs inserted by doctors were removed within 6 months insertion but none of the IUDs inserted by nurses are known to have been removed.	Low
**Both doctors and nurses**	Heath L et al. (2014) ^[^^28^^]^	Eighty-two percent of the respondents had no problems in providing IUD at their first year after training. Of those who had failures in inserting IUD lack of confidence was the main reason for failure	Low
	Goldthwaite LM et al. (2016) ^[^^23^^]^	self-assessed comfortable with knowledge increased (94% at immediate post training and 86% at 6 months follow up; correctly answered knowledge significantly increased); number of HCP reported to place at least one IUD significantly increased from 60% to 81%	Low
	Lewis C et al. (2013) ^[^^15^^]^	After training, the knowledge of IUD increased with average score from 58% to 81%. The mean insertions increased by 5 insertions after training for all participants. The provision of IUD increased more in training sites compared to comparison group.	Moderate
	Postlethwaite D et al. (2007) ^[^^25^^]^	The number of health care providers who reported familiar with IUCD was high in intervention group (75%) compared to control intervention groups (59%). Health care providers from intervention group also reported a more positive attitude towards IUCD and a greater likelihood to recommend IUD for patients compare to comparison group. The IUD ultilization rate was 9.57 per 1000 women with aged 15–44 years old after training compared to 7.35 in group without training (P = 0.02).	Moderate
	Harper CC et al. (2008) ^[^^40^^]^	For most OB-GYNs physicians who received training, only 74% of the provided IUD at their practices. Thirty-two percent of physician assistant and 81% of physicians applied IUCD for patients after training. There is no difference between physicians and nurses in the frequency of IUCD counselling. clinicians who received training were 60% more likely to counsel patients. Training is also significant associated with IUCD provision (OR = 1.8, 95%CI: 1.21 to 2.74). For nurse practitioners who had training, only 43% of them provided IUD at their practices. For all participated nurses, 59% of them applied IUCD to patients after training. There is no difference between physicians and nurses in the frequency of IUCD counselling.	Low
**Residents**	Garcia-Rodriguez JA et al. (2016) ^[^^26^^]^	After training, IUD knowledge were slightly higher in Video group compare to traditional instruction group, but not significant (p>0.05). The score of IUD insertion performance was significant higher in Video group (P<0.05) compare to traditional instruction group.	Moderate
	Amico J et al. (2015) ^[^^33^^]^	The rate of continuation for faculty-inserted devices was higher than trainee-inserted devices. Hazard Ratio was 2.43 for trainees-inserted compare with faculty-inserted. The expulsion rate has no differences between the two groups.	Very low
	Schubert FD et al. (2015) ^[^^34^^]^	After residency training, 87.7% of third-year residents reported inserted at least one IUD; 88.8% of these participants answered at least 4 questions corrected out of 5; 84.6% of residents reported they would like to insert IUD in their future clinics.	Very low
	Romero D et al. (2015) ^[^^19^^]^	After residency training, 84.5% of the respondents felt competent in IUD insertion. The mean procedures of IUD insertion performed by family planning residents who intended to recommend IUCD as an effective contraceptive method were 20 compare to 14 with those not intended.	Very low
	Havilan M et al. (2015) ^[^^35^^]^	Both groups reported increased comfort in IUD insertion. However, 3 months later, participants reported decreased comport and this is no differ in both groups. They also felt increases in self-perceived competence in both groups but this decreased after 3 months.	Low
	Nippita S et al. (2015) ^[^^31^^]^	Both groups felt increased confidence in IUD insertion after training. Self-perceived competency with procedures also improved for both groups. Ninety-three percents of participants in the high-fidelity groups thought the model was valuable compared to 57% in low-fidelity model group.	Low
	Jatlaoui T et al. (2014) ^[^^36^^]^	After training, 99% of the insertions are successful. With 88 women completed at least one contact at the 6 months follow-up, 19.3% had expulsions, 11% were diagnosed with infection. No pregnancies or perforations reported.	Low
	Turk J et al. (2011) ^[^^14^^]^	After training, residents have placed more IUDs than residents at non-LARC sites. Competency scores were significantly higher in all contraception-related procedures including contraception counselling (p < .01), post-abortion insertions (< .01), post-partum IUD insertions (p = .07) compared to non-LARC sites.	Low
	Schreiber CA et al. (2006) ^[^^37^^]^	IUD training was statistically significant associated with higher mean scores of knowledge of IUD. There is evidence that being able to insert an IUD is associated with improved knowledge (p = 0.02). 73% reported that they had received formal training in contraception, only 16% felt able to insert an IUD.	Very low
	Cheng D (1999) ^[^^38^^]^	In FP residency program, no one had managed IUD insertion or removal more than 10 cases while of residents in OB/GYN program, 5% of the participants managed IUD practices more than 10 cases. FP residents reported inadequate training in contraceptive methods. 50% FP residents had never inserted an intrauterine device, 20% of OB residents had never inserted an IUD. Not one FP resident had inserted or fitted more than 10 IUDs, 80% OBs had not inserted more than 10 IUDs	Low

Included studies were conducted in Australia, the UK, the USA, Canada and New Zealand. The IUCD training was provided to family planning physicians (such as obstetricians and gynaecologists), general practitioners, nurses and midwives (Table 2). Canada and the USA have family planning residency programs for healthcare providers; and IUCD training is compulsory for family planning and obstetrics-gynaecology residents [13, 14]. Physician interns, nurse practitioner students and physician assistants in the USA may also receive IUCD insertion training [15]. Family physicians were more likely to receive training in placement of IUCDs compared to medicine physicians (70.4% vs. 9.9%) [16]. Compared to nurse practitioners working in primary care, those working in women's health were more likely to receive IUCD insertion practicum training (12% vs. 66%) [17]. Most of the IUCD training for nurses was conducted in family planning services and clinics [18–21], while for doctors and residents training was primarily in hospitals, medical centres and clinics [22–25].

**Table 2:** Types of IUCD training in high income countries.

Participants of training	Country	Author (year)	Type of IUCD training	Participants of training	Training program	
**Doctors**	AU	Stewart M et al. (2016) ^[^^22^^]^	IUCD insertion training	GPs	Approach standards training, competency-training with IUCD insertions in patients under the experienced doctors’ supervision.	
	USA	Thompson KM et al. (2016) ^[^^24^^]^	Continuing education to clinicians	Clinicians	Four hours continuing medical education with a didactic session on IUCD, a hands-on IUCD insertion practicum for clinicians and counselling role play	
	USA	Luchowski AT et al. (2014) ^[^^13^^]^	Multi-trainings include residency training and continuing education	OBs and GYNs	Didactic and clinical training on IUCD insertion in residency and continuing education recently in the past year	
	USA	Lunde B et al. (2014) ^[^^16^^]^	IUCD placement training	physicians	No specified details about this training program	
	USA	Greenberg KB et al. (2013) ^[^^42^^]^	Family medicine residency training	Adolescent medicine providers	Family planning residency training	
	CA	Phillips SP et al. (2010) ^[^^27^^]^	IUCD insertion workshop	FPs	Three hours skill transfer workshops with peers teaching IUCD insertion, endometrial sampling and pessary fitting	
	USA	Madden T et al. (2010) ^[^^39^^]^	Insertion training in residency program	OBs and GYN physicians	IUCD insertion training during residency or advanced practice core training	
	UK	Moss E et al. (2009) ^[^^41^^]^	Unclear	OBs and GYNs	No specified details about this training program	
	USA	Goodman S et al. (2008) ^[^^32^^]^	Training of insertion, counselling and patient education	clinicians	Focused IUCD training program to reintroduce the Cu-T380a which covers 6 months include instruction in insertion, training in IUCD counselling.	
	UK	Markham WA et al. (2005) ^[^^30^^]^	Sexual health training	GPs	No specified details about this training program	
	NZ	Richardson A et al. (1993) ^[^^29^^]^	General training in IUCD insertion	GPs, FPs, OBs and GYNs	No specified details about this training program	
**Nurses**	AU	Kemeny F et al. (2016) ^[^^18^^]^	Competency-based training program	RNs	Competency-based training program (using pelvic model followed by supervised insertions with Copper IUCD and levonorgestrel-releasing IUCD)	
	USA	Dermish A et al. (2016) ^[^^20^^]^	IUCD insertion training focus on paracervical block and cervical dilatation	NPs and CNMs	Low-cost 2 hrs in-person advanced practice clinicians training focuses on adjunctive method for difficult IUCD insertions	
	USA	Harper CC et al. (2013) ^[^^17^^]^	Insertion training	NPs	Family planning training program, practicum clinical IUCD training, comfortable inserting training of IUCD	
	UK	Andrews GD et al. (1999) ^[^^21^^]^	Nurse specialist training in fitting IUCD	Nurses	Family planning nurses trained to become clinical nurse specialists after a minimum of 2 years’ experience following family planning course with training of IUCD insertion practice under the supervision of Family Planning instructing doctor	
**Doctors and nurses**	UK	Heath L et al. (2014) ^[^^28^^]^	IUCD provision training	NPs and GPs		Training scheme for general practitioners and practice nurses in provision of subdermal implants and IUCD
	USA	Goldthwaite LM et al. (2016) ^[^^23^^]^	Postpartum IUCD training	CNMs and Physicians		Thirty minutes standardized training include didactic, video and hands-on practice sessions which covered insertions at the time of vaginal and caesarean deliveries
	USA	Lewis C et al. (2013) ^[^^15^^]^	Insertion techniques training	NPs, physicians and physician assistants		Six hours IUCD insertions training combined with didactic training with hands-on supervised insertion practice for clinicians
	USA	Postlethwaite D et al. (2007) ^[^^25^^]^	Clinicians peer to peer education	NPs and physicians		IUC insertion training sessions
	USA	Harper CC et al. (2008) ^[^^40^^]^	General training (unclear)	Physicians, physician assistants and NPs		No specified details about this training program
**Residents**	CA	Garcia-Rodriguez JA et al. (2016) ^[^^26^^]^	Video-module instruction	Family medicine residents		Video-module instruction with necessary knowledge and skills to perform an IUCD insertion
	USA	Amico J et al. (2015) ^[^^33^^]^	Family Medicine Residency education	FP residents		Family planning residency programs in an academic family medicine centre
	USA	Schubert FD et al. (2015) ^[^^34^^]^	FP residency training program	FP residents		Family planning residency program
	USA	Romero D et al. (2015) ^[^^19^^]^	Abortion training	Graduate FP residents		The training program is included in their curricula during their Family planning residency programs
	USA	Havilan M et al. (2015) ^[^^35^^]^	Pelvic simulator training models	Interns and NP students		Training of practice on pelvic simulator module with didactic slides and insertion tutorial for practicing
	USA	Nippita S et al. (2015) ^[^^31^^]^	Insertion trainings with pelvic simulator models	Inters and NP students		IUCD insertion training videos before practicing on pelvic simulator models
	USA	Jatlaoui T et al. (2014) ^[^^36^^]^	Insertion training sessions	OBs and GYNs residents		Training sessions include counselling for IUCD, insertion techniques and abdominal ultrasound guidance for fundal placement
	USA	Turk J et al. (2011) ^[^^14^^]^	LARC training in residency program	OBs and GYNs residents		The training program offers technical and financial support to obstetrics–gynaecology residencies for contraception training
	USA	Schreiber CA et al. (2006) ^[^^37^^]^	IUCD training in family medicine residency program	Graduate FP residents		Training in family planning residency
	USA	Cheng D (1999) ^[^^38^^]^	IUCD training in family medicine residency program Maryland	FP and OBs residents		Training in family planning residency

GP = General Practitioner, OB = Obstetrician, GYN = Gynaecologist, FP = Family Planning, RN = registered nurse, NP = nurse practitioner and CNM = Certified nurse-midwife

### Types of IUCD training programs

The majority (90%) of the eligible articles documented the method of training in detail, with most including either continuing education training for qualified healthcare providers or family planning residency training for residents and interns. For qualified healthcare providers, the IUCD training programs were continuing education in IUCD insertion and placement for doctors and/or nurses (Table 2).

There were seventeen (57%) articles that focused on continuing education training programs, which varied by country. In Australia, programs focused on competency-based IUCD insertions, and involved utilisation of pelvic models and clinical practice under the supervision of experienced professionals [18,22]. The training provided to healthcare providers in Canada involved media video modules and peer-to-peer teaching workshops [26,27], while in the UK and New Zealand, programs were described as general training or sexual health training [28,29,30]. In the USA, training focussed on counselling skills as well as IUCD insertion techniques, including hands-on practice under supervision [13,15,17,31].

The training programs also varied according to the different roles of healthcare providers. For registered nurses, the training tended to focus on insertion competency [18,23,28], whereas training for doctors further included counselling skills and knowledge of eligibility criteria for IUCD use [24,27,32].

The remaining 10 studies [14, 19, 26, 30, 33–38] which detailed the method of training explored IUCD training during residency, based in Canada and the USA.

### Effectiveness of IUCD training

#### Improved knowledge and attitudes

There were 5 (17%) studies that reported on improvements in health care providers’ knowledge of and attitudes towards IUCDs after training [15, 17, 23, 26, 37]. For example, 94% of 84 healthcare providers who were trained in IUCD insertions in the postpartum period self-reported increased knowledge after training [23]. The average knowledge score for doctors and nurses in another study improved from 58% to 81% after skills-based IUCD training sessions [15], with increased understanding of how to determine eligibility for IUCD use [17] and more confidence in insertions after the IUCD training program [18,22]. Doctors and nurse practitioners who received peer to peer education reported more familiarity and knowledge about IUCDs compared to those who did not receive any education about IUCDs (75% vs.59%) [25]. Skill-based competency training and didactic sessions also showed positive effects on confidence in IUCD insertion for both doctors and nurses [20,22–23], although one study reported didactic clinical training had no significant impact on improving obstetricians and gynaecologists’ knowledge [13]. Another study reported increased willingness to recommend the IUCD to women with medical conditions such as diabetes, menorrhagia, dysmenorrhea or a history of ectopic pregnancy after completing the training [39]. For family planning residents, the residency training improved their knowledge about contraceptive management in different patient-specific situations [19,26,33,36].

#### Successful IUCD insertions

Altogether, 15 (50%) of the eligible studies focussed on outcomes of IUCD insertions after training. The rate of successful insertions was reported to increase after completion of a training program for both doctors and nurses [18, 20, 22, 27–28]. From a study conducted in the USA, the successful insertion rate in advanced practice healthcare providers (women's health and family practice nurse practitioners, physician assistants and certified nurse midwives) was approximately 4.8 times higher following a short training program focusing on adjunctive methods [20]. In Australia, of 207 insertions undertaken by registered nurses who completed a competency-based training program, 91% of their IUCD insertions were successful and required no assistance from a doctor [18]. For 238 IUCD insertions by doctors in Australia, 89% of the insertions were reported to be successful [22]. A study in the UK reported that 82% of 165 general practitioners felt they had no problems in providing IUCD insertions in the first year after completion of the training scheme [28]. In Canada, the number of successful insertions performed by a family physician increased to 87 insertions after training, compared to 28 successful insertions in those who had no training in the same period [27]. The rate of successful IUCD insertions was also high (99 out of 100) for family planning residents who received training IUCD training [35].

In terms of failed IUCD insertions post-training, some general practitioners reported this as due to lack of confidence [28], while nurse practitioners and certified nurse-midwives reported patient pain was their main cause of failure during the procedure [20].

#### Complications following IUCD insertions

Eight (27%) studies explored adverse effects and complications of IUCD insertion after training. The rates of expulsion, uterine perforation, malpositioning and infection were low in IUCDs inserted by trained doctors and nurses. In a study based in the USA, of 186 insertions performed by nurse practitioners and physician assistants, only one case of perforation and no cases of expulsion were reported [20]. There was no perforation or infection reported during follow up of insertions performed by trained nurses and doctors from studies conducted in Australia [18, 22]. The rate of expulsions and malpositioning was similar in trained nurses and doctors, with 2% of IUCDs expelled and 1% malpositioned in 207 insertions performed by registered nurse [18], compared to 1% expelled and 2% malpositioned of 238 IUCD insertions by doctors [22]. Expulsion rates following IUCD insertions similarly showed no significant difference between faculty staff and trained family planning residents in the USA [32]. However, in another study conducted in the USA, of IUCD insertions attempted by gynaecology and obstetrics residents, 19.3% expulsed and 11% were diagnosed with an infection [35]. Overall, adverse effects and complications including expulsions, perforations, malpositions and infections were rare after training for both doctors and nurses [18–21, 31].

#### Provision of IUCD insertions

Of the included studies, 13 (43%) explored the provision and uptake of IUCDs after training of healthcare providers [15–19, 21, 23, 25, 31, 33, 40–42]. Before training, only 60% of 84 healthcare providers in one study reported inserting at least one IUCD in the postpartum period; this increased to 81% after training [23]. The mean IUCD insertions per month in one setting similarly increased from 28 to 71 after a training program focused at reintroducing the non-hormonal IUCD [31]. Compared to non-participating sites for training, the provision of IUCDs increased by 1.9 to 3.0 times in participating sites [15]. Training for adolescent health care providers also showed increases in provision of IUCDs for young people [42]. Practicum training was associated with 2.4 times provision of IUCDs in nurse practitioners’ later practice compared to their pre-training practice [17].

Residency training also positively affected the provision of IUCD by family planning healthcare providers and residents. Obstetricians and gynaecologists who had residency training were more likely to provide IUCDs for their patients compared to those residents without training [33]. The provision of IUCDs also related to the timing of training: physicians who were trained in IUCD insertion after residency were two times more likely to place IUCDs than those trained during their residency [16].

Most of the studies did not mention the characteristics of women who received an IUCD. Of the few studies that reported this, more parous women than nulliparous women received an IUCD [18–19, 21, 25]. For example, 11% of women who received an IUCD were nulliparous in an Australian study [18], and 22% were nulliparous in another study in the USA [21].

## Discussion

Findings from this systematic review suggest that IUCD training for healthcare providers contributes to improved knowledge and attitudes regarding provision of IUCDs, high rates of successful insertions with low complication rates, and increased provision of IUCDs to women. These changes were similar across the different healthcare providers included in the studies.

Lower uptake of IUCDs can be attributed to low or inaccurate knowledge about this method among healthcare providers and insufficient numbers trained in IUCD insertion [43,44]. Healthcare providers who are not trained to insert IUCDs may be less likely to recommend this contraceptive method, and lack of access to or inability to identify healthcare providers trained in the insertion of IUCDs also impedes uptake [9]. Nurses and midwives play an important role in providing preventative care and counselling in the area of women’s health, especially for women at risk of an unintended pregnancy [45]. Nurse practitioners, registered nurses and midwives are therefore well-placed to provide IUCD insertions and removals. This is occurring in many other countries [16, 32, 33] but is less common in Australia [9].

This systematic review has found that when nurses and midwives are trained in IUCD insertion procedures their procedural outcomes are comparable to doctors [18,21]. Upskilling nurses in this area would offer increased access to IUCDs and increased contraceptive options for women [46,47]. Improving availability and accessibility of the full range of contraceptive options, including IUCDs, is critical to ensure that women have access to their preferred method of contraception.

This is the first systematic review to explore the effectiveness of IUCD training for healthcare providers in high income countries. Findings highlight the beneficial outcomes of IUCD trainings for healthcare providers, which will contribute to promoting provision and uptake of IUCD. However, there are some limitations in this review. We only included studies published in English language journals, which might introduce a bias if the evidence from studies in non-English speaking countries is different to that summarised here. Most of the included studies have a very low to moderate risk of bias due to low participation rates, low follow-up rates and self-reported outcomes. Furthermore, most studies involving both doctors and nurses as participants did not stratify the results to assess whether the training and subsequent insertion outcomes were different, so comparisons are unable to be made. Moreover, the sample sizes of included studies were small, which might lead to a decreased power of detecting important effects. The different country contexts, training settings and clinical scopes of practice, as well as limited sample sizes in some studies, might also affect the generalisability of findings. These issues, as well as the variety of different ways that outcomes of the studies were reported, precluded any formal meta-analysis from being carried out.

## Conclusion

In summary, this review identified different types of IUCD training programs for healthcare providers and how these may contribute to IUCD insertion practices and outcomes in high income countries. Training for healthcare providers was found to be effective in increasing knowledge of IUCDs and successful provision of these. Following training, IUCD insertion outcomes appear to be similar for different healthcare providers. Increasing the number of doctors, nurses and midwives skilled in IUCD insertions will improve access to this method of contraception and allow women greater contraceptive choice.

## Supporting information

S1 Checklist PRISMA checklist. (DOC)
